# Venovenous Extracorporeal Membrane Oxygenation for Negative Pressure Pulmonary Hemorrhage in an Elderly Patient

**DOI:** 10.1155/2015/908157

**Published:** 2015-11-18

**Authors:** Kenichiro Ishida, Mitsuhiro Noborio, Nobutaka Iwasa, Taku Sogabe, Yohei Ieki, Yuki Saoyama, Kyosuke Takahashi, Yumiko Shimahara, Daikai Sadamitsu

**Affiliations:** Traumatology and Critical Care Medical Center, National Hospital Organization, Osaka National Hospital, 2-1-14 Hoenzaka, Chuo-ku, Osaka 540-0006, Japan

## Abstract

The patient in this case report was an 88-year-old male. Acute upper airway obstruction by food led to transient cardiac arrest, and negative pressure pulmonary hemorrhage (NPPH) occurred 1 hour after the foreign body obstruction. Using venovenous extracorporeal membrane oxygenation (ECMO) for severe acute respiratory distress syndrome resulting from NPPH, his respiratory state was recovered and hemoptysis stopped. NPPH is a life-threatening disease, the rapid recognition of which is required to initiate appropriate therapy. Although active hemorrhage might be a contraindication for ECMO, our experience showed this to be an effective treatment option. Moreover, our experience suggests that the application of ECMO to elderly patients should be considered on a case-by-case basis.

## 1. Introduction

Negative pressure pulmonary edema (NPPE) and hemorrhage (NPPH) are uncommon problems resulting from upper airway obstruction [1]. A previous report showed that NPPE and NPPH resolve rapidly with short-term ventilatory support [2]. However, NPPE and NPPH can lead to life-threatening respiratory insufficiency requiring extracorporeal membrane oxygenation (ECMO) [3]. Although active hemorrhage might be a contraindication for ECMO, it is considered for life-threatening acute respiratory distress syndrome (ARDS) when the underlying condition is reversible despite optimal ventilatory support [4]. Age is an important prognosis-related factor [5–7]. However, the application of ECMO to older patients is not contraindicated [8, 9]. This paper presents our experience of successful treatment with venovenous (VV) ECMO for NPPH following foreign body obstruction.

## 2. Case Presentation

The patient was an 88-year-old male. He had chronic heart failure, but his activity of daily living level was independent. When he presented to our hospital for a regular follow-up appointment, he lost consciousness suddenly during lunch at a restaurant in the hospital. Cardiac arrest occurred and bystander cardiopulmonary resuscitation (CPR) was performed by a layperson. Medical staff rushed to the scene, and the initial rhythm at the time of cardiac arrest was nonshockable. Advanced cardiac life support was started immediately. The oral cavity was occluded by rice and a piece of laver; therefore, manual ventilation was impossible. Thus, the foreign body was removed using Magill forceps. The trachea was intubated and spontaneous circulation was resumed after two cycles of chest compression. The patient was taken to the intensive care unit (ICU) for further resuscitation. On admission to the ICU, the patient's blood pressure was 170/105 mmHg, his heart rate was 90 bpm, his peripheral oxygen saturation level was 88% with 10 L/min of oxygen via a bag valve mask, and he was in a coma.

**Figure 1:**
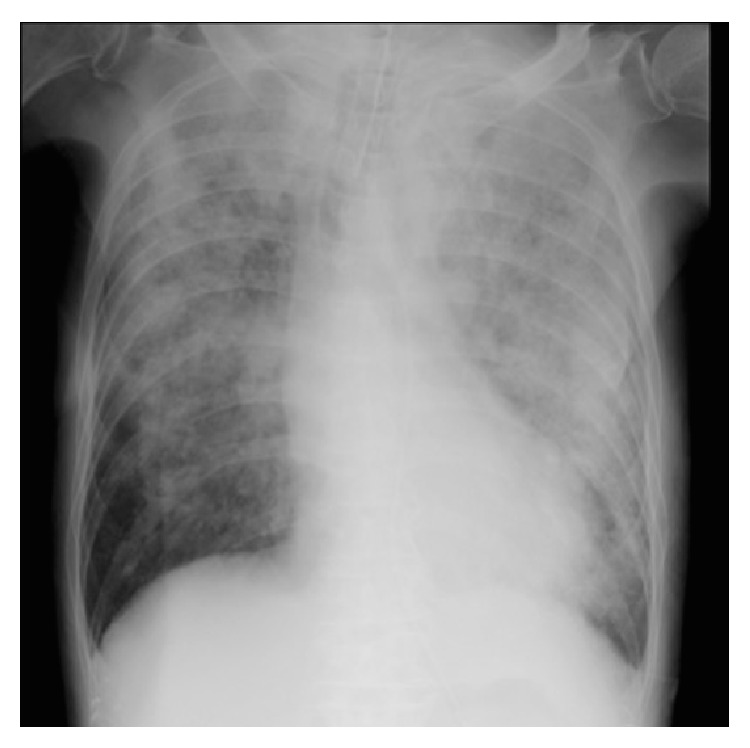
A chest radiograph on admission to the intensive care unit showed diffuse bilateral infiltrates, consistent with pulmonary edema and hemorrhage.

**Figure 2:**
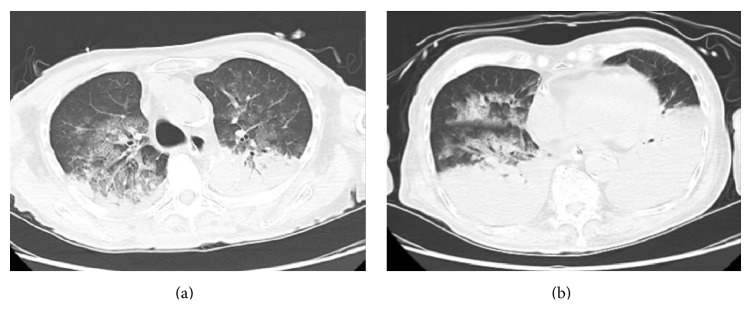
A thoracic computed tomography scan demonstrated bilateral parenchymal consolidation in gravity-dependent areas and ground glass-appearing opacities of lung parenchyma (a). Bilateral atelectasis was observed in the lower lung fields (b).

The patient was responsive to verbal commands immediately after admission to the ICU. However, copious frothy blood-tinged secretions were suctioned from his endotracheal tube 1 hour after the foreign body obstruction. No food was found in the secretions. A chest X-ray showed bilateral infiltrates (Figure 1), compatible with pulmonary hemorrhage. Thoracic computed tomography (CT) showed bilateral parenchymal consolidation in gravity-dependent areas and ground glass-appearing opacities of lung parenchyma (Figure 2). His hemodynamic status was maintained with inotropes, but no abnormality in cardiac function was detected on echocardiography. His respiratory status had promptly deteriorated with hypoxemia, compatible with severe ARDS. The ventilator settings had to be elevated (biphasic positive airway pressure) from 20/10 cmH_2_O to 28/20 cmH_2_O. Fractional inspired oxygen was increased from 60% to 100%, but hypoxemia persisted (PaO_2_ 54 mmHg). Arterial hypercapnia (PaCO_2_ 55 mmHg) also persisted. Therefore, VV ECMO (Capiox emergent bypass system; Terumo Inc., Tokyo, Japan) was used for the treatment of ARDS resulting from pulmonary hemorrhage 5 hours after onset of the latter.

**Figure 3:**
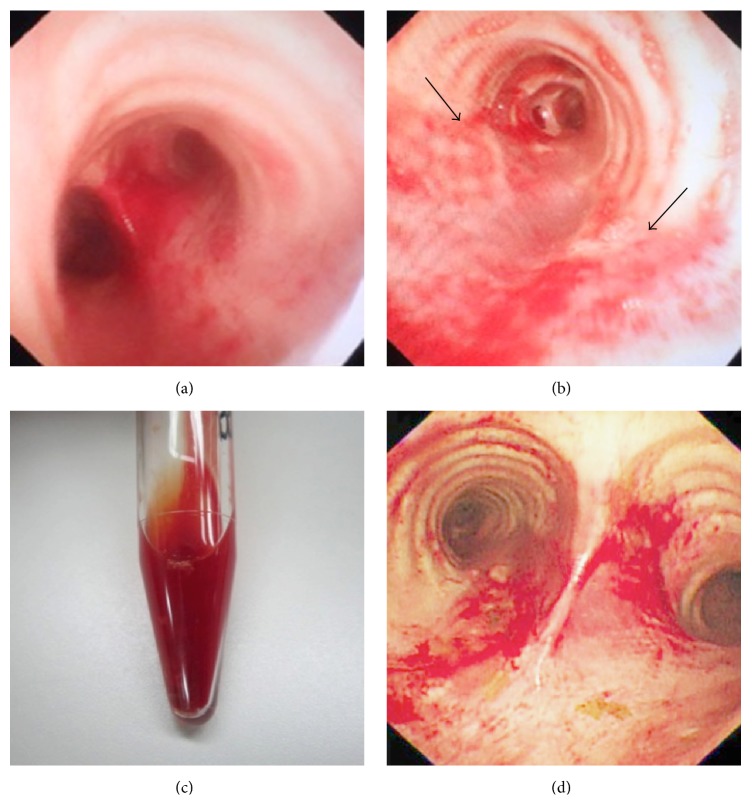
Flexible bronchoscopy on the day of admission revealed fresh blood in the entire tracheobronchial tree (a), and a copious frothy blood-tinged secretion was suctioned form the throat (b, c). Tracheobronchial tree on hospital day 1 (d).

After systemic anticoagulation with 5000 U of heparin delivered intravenously, ECMO was initiated at a flow rate of 2.4 L/min with an 18 Fr drainage cannula in the right femoral vein and a 15 Fr reinfusion cannula in the internal jugular vein. A continuous heparin infusion (400 U/h) was applied to maintain an activated clotting time (ACT) of 160–200 seconds. ACT was measured every 2 hours and a platelet transfusion was required due to gradual progression of thrombopenia. Moreover, red blood cells were transfused to maintain hemoglobin of 12 g/dL and hematocrit of 40%. The ventilator was then set to a lung-protective strategy with a positive end-expiratory pressure of 10 cmH_2_O. On admission and hospital day 1, a flexible bronchoscopy revealed fresh blood in the entire tracheobronchial tree (Figure 3). Cytology was negative for hemosiderin-laden macrophages. Tests for autoimmune markers, including anti-neutrophil cytoplasmic antibodies, were negative. NPPH was diagnosed based on these results. The patient was successfully decannulated after 2 days of ECMO support and weaned off ventilator support within the next 6 days. Hemoptysis decreased progressively and stopped 2 days after ECMO. The only ECMO-related complication was slight thrombus formation in the right jugular and femoral veins, which required continuous heparin infusion after ECMO.

On hospital day 9, the patient was weaned from mechanical ventilation and extubated. However, reintubation was required because of respiratory deterioration due to chronic heart failure related to his underlying illness on hospital day 13. Thereafter, tracheostomy was performed and he was weaned from mechanical ventilation. The length of the ICU stay was 20 days. The patient was alert with no respiratory support or supplementary oxygen and was transferred to a rehabilitation hospital following ICU discharge.

## 3. Discussion

This report raises two important issues. First, NPPH is a reversible disease, but it can be a life-threatening complication of acute upper airway obstruction. Second, ECMO usage is feasible and effective for elderly patients in the clinical setting of pulmonary hemorrhage.

NPPE and NPPH are uncommon problems resulting from upper airway obstruction [1]. Although the mechanisms of these diseases remain uncertain, it has been postulated that increased microvascular pressure and pulmonary capillary permeability, and mechanical disruption of the alveolar capillary membrane, are related to intrathoracic pressure, which results in NPPE and NPPH [10–12]. Acute upper airway obstruction by food led to transient cardiac arrest in our patient. A copious frothy blood-tinged secretion was suctioned from the endotracheal tube 1 hour after the foreign body obstruction was removed. No food was found in the secretions. There were episodes of pulmonary hemorrhage after the upper airway obstruction and negative cytology and autoimmune marker test results, indicating NPPH, not aspiration pneumonia. Several factors may be associated with the onset of NPPH. First, there might be markedly negative intrathoracic pressure during inspiratory effort against airway obstruction. Second, transient cardiac arrest and chest compression during CPR might influence microvascular congestion and hemorrhage. Moreover, capillaries might become fragile in patients of advanced age.

A previous report showed that NPPE and NPPH resolved rapidly with short-term ventilatory support [2]. However, NPPE and NPPH can lead to life-threatening respiratory insufficiency requiring ECMO [3]. In our patient, the ventilator settings had to be raised to maintain the PaO_2_ after the onset of NPPH. However, hypoxemia persisted due to a ventilation-perfusion imbalance resulting from pulmonary hemorrhage and atelectasis. The treatment of NPPE and NPPH is supportive. NPPH is a life-threatening disease, the rapid recognition of which is required to initiate appropriate therapy.

ECMO treatment is considered for life-threatening ARDS when the underlying condition is reversible despite optimal ventilatory support [4]. We initiated ECMO for early-onset NPPH because it was considered reversible. We initially hesitated to initiate ECMO in our elderly patient due to reports showing that the length of mechanical ventilation, severity of multiple organ failure, and immune status of the patients were prognosis-related factors during ECMO. Moreover, age is an important prognosis-related factor [5–7]. However, the application of ECMO to elderly patients is not contraindicated [8, 9]. In our elderly patient, a good result was expected because his underlying condition was considered reversible and he did not have multiple comorbidities.

To safely maintain the ECMO circuit, systemic anticoagulation is required. On the other hand, bleeding is a serious complication of ECMO. The application of ECMO to hemorrhagic patients is not contraindicated and ECMO treatment for hemorrhagic patients—such as those with pulmonary hemorrhage, intracranial hemorrhage, and multiple trauma—has been reported [13–16]. Although the optimal anticoagulation management that minimizes bleeding during ECMO treatment of pulmonary hemorrhage patients is unknown, several methods—such as heparin-free ECMO or the use of nafamostat mesilate as an alternative anticoagulation agent to reduce the risk of bleeding—have been reported [13–15]. In our patient, a continuous heparin infusion was adapted to maintain an ACT of 160–200 seconds. At that time, red blood cell and platelet transfusions were required. Hemoptysis decreased progressively and stopped 2 days after ECMO without uncontrollable bleeding. Changing anticoagulation agents and interventional radiology as a hemostatic option were considered in case of refractory bleeding. ECMO usage was feasible and effective for our elderly patient in the clinical setting of pulmonary hemorrhage.

## 4. Conclusions

In summary, we reported our experience of VV ECMO for NPPH following foreign body obstruction. NPPH is a life-threatening disease, the rapid recognition of which is required to initiate appropriate therapy. Although active hemorrhage might be a contraindication for ECMO, our experience showed this to be an effective treatment option. Moreover, our experience suggests that the application of ECMO to elderly patients should be considered on a case-by-case basis.
